# Reactivity of Health-Related Quality of Life to Perceived Stress: The Buffering Role of Psychosocial Resources in a Longitudinal Study of Adults with and Without HIV

**DOI:** 10.1007/s10880-023-09962-4

**Published:** 2023-05-19

**Authors:** Vanessa B. Serrano, Elizabeth C. Pasipanodya, Jessica L. Montoya, Robert K. Heaton, Dilip V. Jeste, David J. Moore

**Affiliations:** 1 San Diego State University/University of California San Diego Joint Doctoral Program in Clinical Psychology, San Diego, CA USA; 2 Department of Psychiatry, University of California San Diego, HIV Neurobehavioral Research Program, 220 Dickinson Street, Suite B (8231), San Diego, CA 92103 USA; 3 Sam and Rose Stein Institute for Research on Aging, University of California San Diego, La Jolla, San Diego, CA USA; 4 Department of Neurosciences, University of California San Diego, La Jolla, San Diego, CA USA

**Keywords:** Resilience, Social support, Personal mastery, Health, AIDS

## Abstract

People with HIV now have increased longevity; however, their health-related quality of life (HRQoL) still lags significantly compared to people without HIV. Perceived stress negatively impacts HRQoL, whereas psychosocial resources are linked to better HRQoL. This longitudinal analysis aims to explore the buffering role of psychosocial resources on the relationship between HRQoL and perceived stress. Participants (N = 240) included 142 persons with HIV (PwH) and 98 without HIV, *M(SD)* = 50.9(8.1) years. Multilevel models over four study years examined longitudinal relationships between HRQoL (outcome) and perceived stress (predictor) and potential moderation by psychosocial resources (personal mastery, social support, and resilience) by HIV serostatus. Among PwH only, personal mastery (*p* = 0.001), social support (*p* = 0.015), and resilience (*p* = 0.029) were associated with an attenuated effect of perceived stress (less negative slopes) for physical HRQoL over time. Bolstering personal mastery, social support, and resilience may have relevance for improving physical well-being among PwH.

## Introduction

Health-related quality of life (HRQoL) is a multidimensional construct of subjective well-being that encapsulates an individual’s perceptions of their physical, mental, emotional, and social functioning (Guyatt, 1993; Hennessy et al., 1994). There are various correlates of HRQoL dependent on specific HRQoL dimension (e.g., mental HRQoL is more strongly associated with depressive and psychotic symptoms than physical HRQoL, whereas physical HRQoL is more strongly associated with activities of daily living than mental HRQoL), and thus, these constructs tend to be modeled separately (Folsom et al., 2009; Ware, 2000). HRQoL is dynamic and influenced by changes to health status, and it has significant prognostic value as a predictor of negative health outcomes, including risk of future morbidity, hospitalization, and mortality (Brown et al., 2015; Haring et al., 2011). Although the longevity of people with HIV (PwH) has dramatically increased following the advent of antiretroviral therapy, such that average life expectancies among PwH now approach those of the general population (CDC, 2019), both the physical and mental HRQoL of PwH still lag significantly behind those of HIV-uninfected (HIV−) individuals (CDC, 2019; Engelhard et al., 2018; Miners et al., 2014).

HRQoL is sensitive to the influence of psychosocial factors, such as perceived stress. Studies across general and patient populations have linked the experience of perceived stress with reduced HRQoL (Golden-Kreutz et al., 2005; Seo et al., 2018). Among HIV- patient populations, such as individuals living with multiple sclerosis, increases in perceived stress have resulted in decrements to HRQoL longitudinally, even with stable disease characteristics (Wollin et al., 2013). Compared to HIV− individuals, PwH tend to report experiencing more historical and ongoing stressors, including financial concerns, stigma and discrimination, physical or sexual abuse, somatic symptoms, as well as disease burden (Martinez et al., 2012). Psychosocial resources (e.g., social support, self efficacy) also tend to be lower among PwH than HIV- individuals (Li et al., 2021) and are influenced by HIV-specific stressors (e.g., HIV stigma; Zhao et al.). Notably, psychosocial resources and coping strategies have been found to be protective of well-being and linked to better HRQoL in the context of stress among both PwH and HIV- individuals (Fang et al., 2015; Gibson et al., 2011). In particular, personal mastery (i.e., the extent to which individuals feel in control of factors that significantly influence their lives; Pearlin & Schooler, 1978), resilience (i.e., the ability to adapt successfully to stress, trauma and adversity; Connor & Davidson, 2003), and social support (i.e., the quality of interactions across an individual’s social network; Pachana et al., 2008) have all been reported to moderate the relationship between stress and HRQoL in cross-sectional investigations of clinical populations (Abshire et al., 2018; Gibson et al., 2011; Li et al., 2019). Nevertheless, the combination of daily life stressors, HIV-specific stressors, and fewer psychosocial resources relative to HIV- individuals, may weaken the protective effects of psychosocial resources for PwH. Taken together, it is of great importance to examine potential psychosocial moderators of the longitudinal association between perceived stress and HRQoL among PwH and HIV− individuals.

Personal mastery is a coping resource that incorporates self-efficacy and locus of control (Pearlin & Schooler, 1978). Among patient populations, self-efficacy is negatively associated with depression and anxiety (van Diemen et al., 2017), and positively associated with medication management and quality of life (McCoy et al., 2016; Tuluce & Kutluturkan, 2018). Longitudinally, supporting personal mastery through intervention has been associated with increased HRQoL (Andenæs et al., 2012), although this relationship been less explored among PwH. An internal locus of control has been associated with higher physical and mental HRQoL among PwH (Mostafavian et al., 2018). Taken together, personal mastery has been associated with psychological HRQoL (Emlet et al., 2017) and may play a role in the prevention of physical deficits in HIV, such as frailty (Rubtsova et al., 2018). Furthermore, personal mastery has been found to significantly moderate the relationship between stress and mental HRQoL among PwH (Gibson et al., 2011). Because of the health benefits personal mastery may confer to PwH, as supported by between-person associations, the longitudinal implications of personal mastery among aging PwH should be explored. Considering how personal mastery relies on an individual’s culmination of life experiences and development of self-efficacy, it is also of interest to examine if the hypothesized moderating effect of personal mastery on the longitudinal relationship between perceived stress and HRQoL differs by age.

Resilience has also been reported to be associated with positive health outcomes in the general population, including successful aging, lower depression, and longevity (MacLeod et al., 2016; Moore et al., 2013; Oppenheim et al., 2018). Researchers have proposed that resilience may regulate the physiological stress response, resulting in better health-related outcomes across middle-aged and older adults (Gaffey et al., 2016; Lehrer et al., 2020). In the context of older PwH, resilience has been positively associated with perceived successful aging (Fumaz et al., 2015), and may reduce the negative influence of life stress on physical, emotional, and functional well-being (Fang et al., 2015). However, a majority of studies on resilience among PwH examined associations with HRQoL and stress cross-sectionally, which precludes cause-effect inferences (Fang et al., 2015; Fumaz et al., 2015), as well as the potential for resilience to be protective over the course of the lifespan. Thus, it is of interest to examine whether resilience may moderate the relationship between perceived stress and HRQoL as PwH advance in age.

In both general and clinical populations, social support has been found to lessen the negative effect of stress on HRQoL (Abshire et al., 2018; Hsieh & Tsai, 2019). Specific to PwH, longitudinal effects of social support among have been found to improve health behaviors such as medication adherence and healthcare utilization, which may directly impact HRQoL (Chandran et al., 2019). In particular, high emotional social support has been associated with better perceived health, higher life satisfaction (Strine et al., 2008), as well as better physical and mental HRQoL (Arabyat & Raisch, 2019), and may be more predictive of subjective well-being than instrumental support (Morelli et al., 2015). Considering the salutary effect of emotional support on HRQoL, examining the influence emotional social support may have on the relationship between perceived stress and HRQoL may underscore the significance of this particular social support resource as older PwH age.

Although there is much literature on the effect of perceived stress on HRQoL, and the moderation of perceived stress by personal mastery, resilience, and social support has been previously examined, little research has explored these relationships longitudinally. Additionally, previous work examining the associations of perceived stress with HRQoL and moderation by positive psychosocial resources has not evaluated these associations among individuals who are aging with HIV (Abshire et al., 2018; Arabyat & Raisch, 2019; Gibson et al., 2011). Therefore, the current study was designed to investigate the relationship between perceived stress and HRQoL over time, and to probe for moderators of this association. It was hypothesized that, accounting for relevant covariates, (i) having increases in perceived stress would result in decreasing HRQoL for PwH and HIV− individuals, (ii) having greater personal mastery, resilience, and emotional social support at baseline would buffer the negative effect of increases in perceived stress on HRQoL over time, and (iii) having HIV would weaken the degree to which personal mastery, resilience, and social support moderate the negative effect of perceived stress on HRQoL, whereas older age would strengthen the hypothesized moderating effects of these psychosocial resources.

## Method

### Participants and Procedure

Participants were 142 PwH and 98 HIV− individuals who participated in the five-year multi-dimensional successful aging among HIV-infected adults study conducted at a university-based HIV research center [name redacted for blind review]. Participants were recruited from ongoing research studies, a university medical center clinic for HIV care (i.e., participant recruitment staff were stationed in the lobby area), and via flyers and presentations at community-based meetings. Previously described in [redacted for blind review], eligible participants were: (1) aged 36–65 years at baseline, (2) fluent in English, and (3) able to provide informed consent. Exclusion criteria were (1) neurologic condition other than HIV known to impact cognitive functioning (e.g., stroke), (2) psychotic disorders, and (3) positive urine toxicology on the day of testing for illicit substances other than cannabis. By design, participants were recruited into roughly equivalent sample sizes of age cohorts by decade at baseline (i.e., ages 36–45, 46–55, and 56–65 years). The study received approval by the local Institutional Review Board. Participants provided written, informed consent.

### Measures

Enrolled participants were followed for up to 5 years, with comprehensive in-person visits every other year. In between comprehensive in-person visits, participants completed some questionnaires by mail.

### Demographic and Clinical Variables Collected at Baseline

In addition to standard sociodemographic information collected at baseline (e.g., age, education, gender, race/ethnicity, employment status, and socioeconomic status), the following neuromedical and psychiatric information was collected.

#### Neuromedical Assessment

PwH and HIV− individuals received a comprehensive neuromedical evaluation. Among PwH, the following HIV disease characteristics were collected by self-report, collected lab value, and/or medical record review: CD4 + T cell counts (nadir and current), estimated duration of HIV disease, AIDS status, and current antiretroviral therapy (ART) regimen.

#### Hollingshead Index

The Hollingshead Index is a measure of socioeconomic status (SES) based on education, occupation, sex, and marital status to determine a family’s composite SES (Hollingshead, 1975). Hollingshead Index raw scores range from 8 to 66, with higher scores reflecting higher SES.

#### Psychiatric and Substance Use Disorders

The Composite International Diagnostic Interview was administered (CIDI, v2.1; World Health Organization, 1997) to assess for lifetime and current major depressive disorder (MDD) and substance use disorders (abuse or dependence) based on the fourth edition of the Diagnostic and Statistical Manual of Mental Disorders (American Psychiatric Association, 2000).

#### Negative Life Events

The incidence of eleven negative life events (e.g., death of a spouse or partner and major accidents, disasters, muggings, unwanted sexual experiences, robberies or similar events), as well as if a change in residence had occurred in the last year, were dichotomously reported using the Women’s Health Initiative Life Events scale (Berkman & Syme, 1979; Matthews et al., 1997). The scale also measured the degree to which an event upset the individual if it did occur, ranging from 2 (not too much) to 4 (very much). Lower scores suggest that events did not occur or had little emotional effect on the individual.

## Variables Examined Longitudinally

### The Following Variables were Collected Annually for up to Five Years

#### Dependent Variable of Interest: Health-Related Quality of Life (Annually)

The Medical Outcomes Study Short Form-36 (SF-36) consists of eight primary subscales (36 items) capturing both physical and mental health-related QoL (Ware, 2000). Mental and physical health summary score reliability estimates generally exceed 0.90 (Ware et al., 1994), and has been used widely among subpopulations of PwH (Cooper et al., 2017). For the physical QoL scale, participants selected the degree to which they are presently limited when completing various physical activities, such as climbing one flight of stairs. Responses ranged from 1 (limited a lot) to 3 (not limited at all). The mental health QoL scale includes items such as, “Have you felt so down in the dumps that nothing could cheer you up?” Item responses used six-point Likert-type scales ranging from 1 (all of the time) to 6 (none of the time). The average score for the SF-36 is 50. The intraclass correlation coefficient (ICC), a measure of the amount of variability due to clustering (with a value of 1 indicating all responses within a cluster are identical), indicated interdependence in observations (due to repeated measurement of the same individuals) and substantial within-person variability for mental health QoL (ICC = 0.47) and physical health QoL (ICC = 0.49; Killip et al., 2004).

#### Independent Variable of Interest: Perceived Stress (Annually)

The 10-item version of the Perceived Stress Scale (PSS) was used to measure perceptions of stress in the last month (Cohen et al., 1983). Cronbach’s alpha of the 10-item scale has been found to be > 0.70 in several studies administering the scale among clinical populations, including PwH (Lee, 2012). The scale includes items such as, “How often have you felt that you were unable to control the important things in your life?” and “How often have you felt difficulties were piling up so high that you could not overcome them?”. Participants were asked to indicate how frequently they experienced each of the 10 items on a 5-point Likert-type scale ranging from 0 (never) to 5 (often). Scores range from 0 to 40, with higher scores being indicative of higher perceived stress. The ICC indicated substantial within-person variability for perceived stress scores (ICC = 0.70).

### Baseline Variables of Interest: Hypothesized Moderators

#### Personal Mastery (Baseline)

The Pearlin Mastery Scale was used to capture the degree to which participants consider themselves to be in control of life outcomes (Pearlin & Schooler, 1978). Scores for the seven-item scale range from 7 to 28, with higher scores being indicative of higher personal mastery. The scale includes items such as “What happens to me in the future mostly depends on me”, and has been considered to be reliable among patient populations (omega > 0.8); Lim et al., 2022.

#### Resilience (Baseline)

The 10-item Connor-Davidson Resilience Scale is a self-report capturing how well an individual perceived their recovery from stressful events, tragedy, or trauma, with a psychometric analysis indicating good reliability (alpha value of 0.85) (Campbell-Sills & Stein, 2007; Connor & Davidson, 2003). Respondents rated each item on a 5-point Likert scale ranging from 0 (not true at all) to 4 (true nearly all of the time), resulting in a total score ranging from 0 to 40, with higher scores being indicative of higher resilience. Sample items include “I am able to adapt to change” and “I believe I can achieve my goals.”

#### Social Support (Baseline)

Considered a category of social support, the 7-item Emotional Support Scale was used to measure the frequency and availability of emotional support Cronbach’s alpha = 0.60. (Seeman et al., 2001). Emotional support item scores range from 0 to 3, with higher total scores indicating more emotional support. Sample items include “How often do your spouse, children, close friends and/or relatives make you feel loved and cared for?” and “How often do your spouse, children, close friends and/or relatives give you advice or information about medical, financial, or family problems?”.

### Data Analysis

#### Statistical Analyses

Analyses were conducted in Mplus version 8.4 and JMP ® Pro 14.0.0 (Copyright^©^ 2018 SAS Institute, Inc.). Comparisons of demographic, medical comorbidities and anthropometric measurement, medication use, psychiatric characteristics/diagnoses between PwH and HIV- individuals were performed using two-tailed *t* tests for continuous variables or Pearson’s chi-squared test for nominal variables. For these group comparisons, effect sizes (i.e., odds ratios or Hedge’s *g*) were calculated. Due to the correction factor used in the calculation of Hedge’s *g,* it is considered to have less bias than Cohen’s *d* (National Institute of Standards and Technology, 2018). Multiple sociodemographic and biomedical variables that have been associated with HRQoL in existing literature; thus, we screened variables listed in Table 1 as potential covariates to include in the multilevel models if they were associated with either mental HRQoL or physical HRQoL at a critical α = 0.10 (Althoff et al., 2016; Low & Molzahn, 2007; Machon et al., 2017).

**Table 1:** Participant demographics and HIV characteristics by serostatus

	HIV–negative (n = 98)	PwH (n = 142)	p value	Effect size
Demographic				
Age (years), mean (SD)	51.1 (7.6)	50.8 (8.4)	.75	g = .04
Education (years), mean (SD)	15.1 (2.2)	14.3 (2.6)	.01*	g = .3
Sex (male), n (%)	67 (68.4%)	122 (85.9%)	.001*	OR = 2.8
Proportion non–Hispanic white, n (%)	67 (68.4%)	78 (54.9%)	.04*	OR = 5.6
Employment (unemployed)	26 (26.5%)	92 (64.8%)	< .001*	OR = 5.1
Socioeconomic status (Hollingshead index)	49.2 (8.9)	40.2 (12.6) ^f^	< .0001*	g = .83
HIV Characteristics				
Duration of HIV infection (years), mean (SD)	–	15 (9.7)	–	–
Proportion with AIDS diagnosis, n (%)	–	78 (32.5%)	–	–
On antiretroviral therapy, n (%)	–	136 (95.8%)	–	–
Detectable plasma viral load, n (%)	–	10 (7%)	–	–
Nadir CD4, median (IQR)	–	200 (58–363.3)	–	–
Current CD4, median (IQR)	–	628.5 (432–854.5)	–	–
Medical Comorbidity				
Diagnosed with diabetes, n (%)	7 (7.1%)	16 (11.3%)	0.3	OR = 1.7
Diagnosed with hypertension, n (%)	19 (19.4%)	59 (41.6%)	< .001*	OR = 3
Diagnosed with hyperlipidemia, n (%)	22 (22.5%)	53 (37.3%)	.01*	OR = 2.1
Metabolic comorbidities, mean (SD)^a^	0.16 (0.26)	0.3 (0.3)	< .001*	g = .5
Neurocognitive & psychiatric disorders				
Global cognitive deficit score	0.27 (.32)	0.44 (.52)	**.003**	g = .39
Lifetime psychiatric disorder, n (%)^b^	20 (20.4%)^d^	79 (55.6%)^e^	< .001*	OR = 5.4
Lifetime major depressive disorder, n (%)	19 (19.4%)^d^	70 (51.5%)^e^	< .001*	OR = 4.4
Lifetime substance use disorder, n (%)^c^	32 (32.7%)^d^	60 (44.1%) ^e^	.08	OR = 1.6
Lifetime alcohol use disorder, n (%)	29 (29.6%)^d^	68 (50%)^e^	.002*	OR = 2.4
Lifetime methamphetamine use disorder, n (%)	0 (0%)^d^	44 (32.4%)^e^	< .001*	–
Lifetime cocaine use disorder, n (%)	14 (14.3%)^d^	30 (22.1%)^e^	.13	OR = 1.7
Lifetime cannabis use disorder, n (%)	17 (17.4%)^d^	32 (23.5%)^e^	.25	OR = 1.5
Perceived stress (hypothesized independent variable)				
Perceived Stress, mean (SD)	10.3 (6.4)	14.8 (8.3)	< .001*	g = .59
Hypothesized psychosocial moderators				
Personal Mastery, mean (SD)	23.4 (3.6)	21.5 (4.4)	< .001*	g = .47
Emotional social support, mean (SD)	3.6 (9.8)	2.4 (.8)	.14	g = .19
Resilience, mean (SD)	34.3 (11.2)	29.6 (7.5)	< .001*	g = .51

^a^Average number of metabolic comorbidities (i.e., diabetes, hypertension, or hyperlipidemia)

^b^Variable included major depressive disorder, bipolar disorder I and II, and lifetime dysthymic disorder

^c^Main variable included other substance use disorders (e.g., hallucinogens, PCP, inhalants, etc.), but the proportion of individuals meeting those disorders was less than 15% and therefore not included in the table

^d^Hedge’s g interpreted as 0.2 = small, 0.5 = medium, 0.8 = large

^e^n = 98

^f^n = 136

^g^n = 139

Multilevel modeling was used to examine the influence of perceived stress on HRQoL and the moderation of this effect by psychosocial resources measured at baseline. Random slopes were used to model the average within-person relationship between perceived stress and HRQoL over time. The moderating influences of baseline personal mastery, resilience, and emotional support on the longitudinal relationship between stress and HRQoL were separately examined as cross-level interactions while controlling for relevant covariates. Considering previous literature describing differences in HRQoL outcomes between PwH and HIV− individuals and to understand how psychosocial moderators may differentially influence the longitudinal association between perceived stress and HRQoL by HIV status, we evaluated separate multilevel models for each of the HIV status groups (i.e., separate models for PwH and HIV− individuals). Additionally, we evaluated separate multilevel models for each of the age cohorts (i.e., ages 36–45, 46–55, and 56–65 years at baseline) while controlling for HIV status to elucidate whether the hypothesized moderating influences of baseline personal mastery, resilience, and emotional support on the association between perceived stress and HRQoL differed by age cohort.

## Results

As shown in Table 1, HIV serostatus groups were comparable in age, with PwH being, on average, 50.8 years of age (*SD* = 8.4), and HIV− individuals 51.1 years of age (*SD* = 7.6). PwH were more likely to be male (85.9% male compared to 68.4% male; *χ*^2^ (1, *N* = 240) = 10.7, *p* = 0.001). The HIV− group had significantly more years of formal education [*M* = 15.1 years, *SD* = 2.2 compared to *M* = 14.3 years, *SD* = 2.6; *t*(238) = 6.7, *p* = 0.01]. Over half of the HIV- participants (68.4%) as well as 54.9% of PwH were non-Hispanic white [*χ*^2^ (1, *N* = 240) = 4.4, *p* = 0.04)].

Among PwH, the vast majority were currently on ART (95.8%) and had undetectable plasma viral loads (83.8%). The median duration of time living with HIV was 15 years (IQR = 5.2–24.0). Nearly a third (32.5%) reported receiving a diagnosis of AIDS.

PwH reported more perceived stress at baseline than HIV- individuals (*M* = 14.8, *SD* = 8.3, versus *M* = 10.3, *SD* = 6.4, *t*(237) = 4.5, *p* < 0.0001). On all items of the perceived stress scale, PwH reported significantly higher scores (i.e., more perceived stress) than their HIV− counterparts. PwH also reported significantly lower personal mastery at baseline compared to HIV− individuals (*M* = 21.5, *SD* = 4.4, versus *M* = 23.4, *SD* = 3.6; *t*(236) = -3.6, *p* < 0.001), as well as significantly lower resilience (PwH: *M* = 29.6, *SD* = 7.5, versus *M* = 34.3, *SD* = 11.2; *t*(238) = − 3.9, *p* = 0.0001). There were no significant differences between social support scores among PwH and HIV− individuals at baseline.

### Relationship between Perceived Stress and HRQoL

Controlling for relevant covariates (i.e., age, education, SES, employment, global cognitive deficit score, and medical/psychiatric comorbidities), mental and physical HRQoL significantly decreased over time among PwH (*B* = − 0.57, *p* = 0.013 and *B* = − 0.54, *p* = 0.008, respectively; Table 2). The random slope of the average within-person relationship between perceived stress and mental HRQoL was negative and significant (*B* = − 1.222, *p* < 0.001), suggesting decreasing mental HRQoL with increases in perceived stress. The random slope of the average within-person relationship between perceived stress and physical HRQoL was not statistically significant (*B* = − 0.16, *p* = 0.09).

**Table 2:** Multilevel model examining the relationship between stress and HRQoL among PLWH and individuals living without HIV infection

	HIV–uninfected				PwH			
	Mental HRQoL		Physical HRQoL		Mental HRQoL		Physical HRQoL	
	Estimate (SE)	*p* value	Estimate (SE)	*p* value	Estimate (SE)	*p* value	Estimate (SE)	*p* value
Within–person fixed effect								
Time	0.11 (0.18)	0.52	− 0.19 (0.25)	0.44	− 0.57 (0.23)	0.013	− 0.54 (0.2)	0.008
Random slope								
Perceived stress	− 0.57 (0.12)	< 0.001	− 0.22 (0.12)	0.06	− 1.22 (0.12)	< 0.001	− 0.16 (0.09)	0.09
Between–person fixed effects								
Age	–	–	− 0.23	0.007	–	–	− 0.05	0.67
Education	− 0.34 (0.39)	0.38	− 0.8 (0.48)	0.09	− 0.66 (0.63)	0.3	0.16 (0.65)	0.81
Socioeconomic status^a^	− 0.03 (0.104)	0.81	0.29 (0.15)	0.05	− 0.09 (0.14)	0.5	0.17 (0.13)	0.19
Employment	1.63 (2.03)	0.42	2.92 (1.83)	0.11	0.46 (2.07)	0.83	6.64 (2)	0.001
Global cognitive deficit score	− 0.37 (1.7)	0.83	− 2.62 (1.98)	0.18	− 0.81 (2.24)	0.72	− 0.28 (2.53)	0.91
Metabolic comorbidities^b^	− 1.01 (0.95)	0.29	− 2.26 (1.04)	0.03	–0.39 (1.18)	0.74	− 0.81 (1.2)	0.5
Lifetime psychiatric disorder	− 1.49 (1.93)	0.44	− 4.14 (2.35)	0.08	− 11.25 (2.06)	< 0.001	− 1.66 (2.02)	0.41
Lifetime substance use disorder	− 0.87 (2.1)	0.68	–	–	− 3.22 (2.27)	0.16	–	–

Age was significantly associated with physical HRQoL but not mental HRQoL, and thus excluded from the mental HRQoL model. Lifetime substance use disorder was significantly associated with mental HRQoL but not physical HRQoL and thus excluded from the physical HRQoL model

^a^Socioeconomic status was assessed with the Hollingshead Index

^b^Average number of metabolic comorbidities (i.e., diabetes, hypertension, or hyperlipidemia)

Among HIV− individuals, physical and mental HRQoL were stable over time, controlling for relevant covariates (i.e., age, education, SES, employment, global cognitive deficit score, and medical/psychiatric comorbidities). As found among PwH, the random slope of the average within-person relationship between stress and mental HRQoL was also negative and significant (*B* = − 0.6, *p* < 0.001), suggesting decreasing mental HRQoL with increases in perceived stress. The random slope of the average within-person relationship between perceived stress and physical HRQoL was not significant (*B* = − 0.22, *p* = 0.06).

### Buffering Role of Psychosocial Resources on HRQoL by HIV Status and Age

**Fig. 1:**
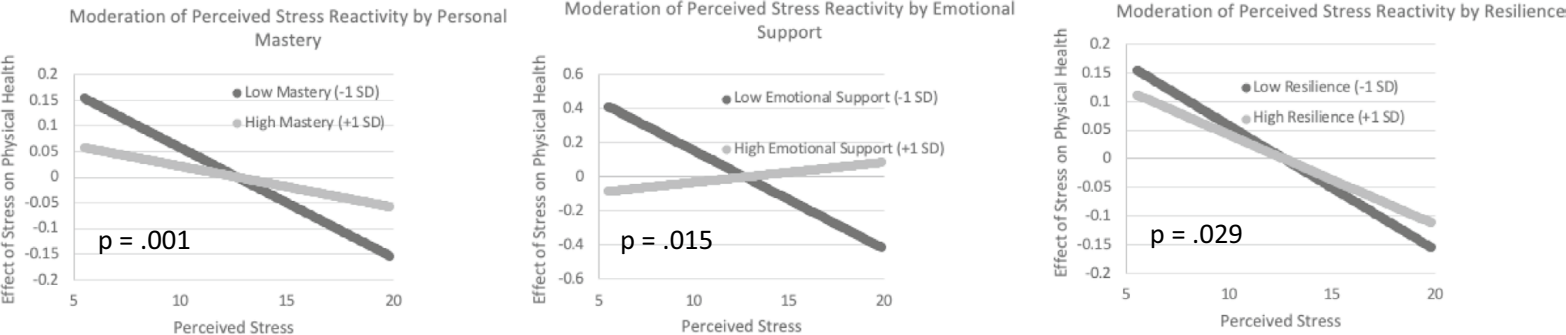
Personal mastery, resilience, and emotional social support moderate the relationship between perceived stress and physical HRQoL among PwH

Personal mastery did not influence the degree to which perceived stress impacted either mental or physical HRQoL across HIV− individuals. However, as shown in Fig. 1, among PwH, higher levels of personal mastery were associated with an attenuated association (i.e., less negative slope) in the relation between physical HRQoL and perceived stress (*B* = 0.048, *p* = 0.001). On the other hand, no moderating effect of personal mastery was found between perceived stress and mental HRQoL for PwH. When examined by age cohort (controlling for HIV status), higher levels of personal mastery were also associated with an attenuated association (i.e., less negative slope) in the relation between physical HRQoL and perceived stress among individuals aged 36–45 (*B* = 0.064, *p* = 0.003). Higher levels of personal mastery also were associated with a slightly less negative relationship between perceived stress and mental HRQoL among individuals aged 56–65 (*B* = 0.045, *p* = 0.058).

Among HIV- individuals, resilience did not influence the degree to which perceived stress impacted either mental or physical HRQoL. Among PwH, however, higher levels of resilience were associated with an attenuated association in the relation between physical HRQoL (but not mental HRQoL) and perceived stress (*B* = 0.022, *p* = 0.029; Fig. 1). When examining the influence of age, however, the moderating effect of resilience was not found to differ by age cohort.

Emotional social support did not influence the degree to which perceived stress impacted either mental or physical HRQoL among HIV- individuals. Among PwH, higher levels of resilience were associated with an attenuated association in the relation between physical HRQoL (but not mental HRQoL) and perceived stress (*B* = 0.248, *p* = 0.015; Fig. 1). The moderating effect of social support did not significantly differ by age cohort.

## Discussion

In this longitudinal study we examined psychosocial moderators (i.e., personal mastery, resilience, and emotional social support) of perceived stress and HRQoL in middle-aged to older people with and without HIV. Although previous research has examined psychosocial resources as protective factors of HRQoL among PwH, this relationship has not been thoroughly examined 1) longitudinally across three age decades (ages 36–45, 46–55, and 56–65 years) and 2) in a well-characterized research cohort consisting of PwH and HIV- individuals, as this study accomplished. Consistent with our first hypothesis, greater perceived stress was associated with lower physical and mental HRQoL over time for PwH and HIV− individuals. Our second hypothesis that psychosocial resources would buffer the longitudinal relationship between perceived stress and HRQoL was partially supported such that this relationship was found for mental but not physical HRQoL, and among PWH but not HIV- individuals. Finally, our third hypothesis that the degree of moderation would be (1) lower among PwH, and (2) stronger among individuals of older age was not supported. Instead, we found (1) strong moderating effects of psychosocial resources only among PwH, and (2) found the moderating effect of personal mastery between perceived stress and physical HRQoL (controlling for HIV status) to be significant among the youngest age cohort (i.e., 36–45 age cohort).

Previous work has examined the salutary effects of psychosocial resources on HRQoL in the context of perceived stress, with findings that the negative effects of perceived stress on HRQoL can be attenuated by perceptions of personal mastery, emotional social support, and resilience (Fang et al., 2015; Gibson et al., 2011; Li et al., 2019). However, the moderating role of personal mastery, emotional social support, and resilience has not been examined among older individuals of mixed HIV-serostatus, precluding a broader understanding of relationships across HIV serostatus and among aging adults. Thus, this study was designed to examine the effects of perceived stress on HRQoL by HIV serostatus among aging individuals living with and without HIV and to examine the influence of greater personal mastery, higher emotional social support, and higher resilience on the relationship between perceived stress on HRQoL.

Across previous studies of PwH, an inverse relationship has been observed between perceived stress and HRQoL (Persons et al., 2010). Our work further supports these findings, and also extended this line of research by exploring within-person associations among older PwH. Although we did not observe the hypothesized relationship between perceived stress and HRQoL over time among HIV- individuals, this may be due to previously documented reduced perceived stress from mid-life onward (e.g., resulting from reduced workload and familial responsibilities as individuals age) within the general population (Hedgeman et al., 2018; Warttig et al., 2013), and thus having a less negative impact on HRQoL. In summary, our finding of an inverse relationship between perceived stress and HRQoL among PwH but not HIV− individuals possibly may be explained by the increased stress HIV imposes on older PwH (Fang et al., 2015).

Partially consistent with our hypotheses, personal mastery had a moderating effect on the relationship between perceived stress and HRQoL among PwH. However, this effect was only observed for physical HRQoL, such that personal mastery did not moderate the relationship between perceived stress and mental HRQoL. These findings contrast those of Gibson et al. (2011), who found that personal mastery moderates the relationship between stress and mental HRQoL, but not physical HRQoL. Although our differing findings for mental HRQoL are unclear, our physical HRQoL findings suggest that high levels of psychosocial resources, such as personal mastery, may compensate for the negative influence of perceived stress on HRQoL. Contrary to our hypotheses, personal mastery did not moderate the relationship between perceived stress and either type of HRQoL among HIV- individuals. Although there are various studies examining personal mastery and perceived stress among HIV− older adults, none have examined these variables in relation to HRQoL. We do know that HRQoL is strongly influenced by health status and medical conditions (Hodek et al., 2009). Thus, perhaps it is the presence of a health condition such as HIV and its comorbidities (Table 1) that makes personal mastery particularly salient between perceived stress and HRQoL, potentially explaining why personal mastery had very little effect among HIV− older adults.

We further explored the role of personal mastery on the relationship between perceived stress and HRQoL by examining age cohorts. Consistent with our conceptualization of personal mastery (i.e., highly related with self-efficacy), and prediction that personal mastery may increase in middle age due to more life experience and further opportunity for demonstration of self-efficacy, we found personal mastery to attenuate the associations between (1) perceived stress and physical HRQoL for individuals aged 36–45, and (2) perceived stress and mental HRQoL among individuals aged 56–65. This difference in findings across age cohorts may be explained by a lower likelihood of physical health conditions being common among individuals aged 36–45 compared to older age cohorts. Further, considering that individuals aged 56–65 may have had longer time to develop personal mastery than younger age cohorts, this may explain a greater impact of their personal mastery being protective of mental HRQoL.

Although greater emotional social support was associated with greater physical HRQoL among PwH, contrary to our hypotheses, emotional social support moderated the associations between perceived stress and physical but not mental HRQoL in this sample. Furthermore, among HIV− participants, emotional social support did not buffer either physical or mental HRQoL. Our findings are similar to those of Abshire et al (2018), who found that social support moderated the relationship between perceived stress and overall HRQoL among patients living with heart failure, however, their study did not separate mental and physical HRQoL.

Our findings for HIV− participants, and in part for PwH, conflict with research suggesting that social support may serve as a buffer between stress and both physical and mental health (Cohen & Wills, 1985; Wilcox, 1981). Although the reason for this discrepancy is unclear, the health outcomes used in existing social support research generally rely on dichotomous checklists assessing for the presence of mental and physical health conditions (Cohen & Wills, 1985), which differs somewhat from HRQoL. Thus, to our knowledge, the present study provides the first examination of both physical and mental health HRQoL and how social support may attenuate the negative effect of perceived stress.

We found resilience to buffer the relationship between perceived stress and physical, though not mental, HRQoL among PwH. Again, our study was the first to examine this particular model, making comparisons of these specific findings difficult. Our findings are similar to another clinical population study in which resilience buffered stress on quality of life, however, in the other study, quality of life was not separated into physical and mental health domains (Li et al., 2019). Although we did not find any moderating effect of resilience on HRQoL for HIV− individuals, a possible explanation may be the same as described for personal mastery: in the context of HRQoL, perhaps it is the presence of a health condition such as HIV that makes resilience particularly salient between perceived stress and HRQoL, explaining why resilience had very little effect among HIV− older adults.

This work has several limitations. First, our study participants were predominantly white and male, potentially limiting generalization to the wider population of aging adults. Second, all predictor and outcome data were based on self-report, which is influenced by participant response styles and accuracy of self-perceptions. Third, although some of the psychosocial moderators, [e.g., resilience (Jacelon, 1997), personal mastery (Hovenkamp-Hermelink et al., 2019)] are considered to have trait-like characteristics, and thus may have intraindividual changes, assessment at baseline precluded examining relationships between fluctuations in the moderators and HRQoL. Future work should examine the extent of stability versus change in these moderators, particularly in the context of intervention. Fourth, there are few studies that have examined this particular set of psychosocial resources, making comparisons of our findings to other studies difficult. Additionally, to provide stronger support of the direction of cause and effect, future work should also consider examining these associations in lagged analyses. Future work may also consider sampling a more diverse and larger sample of aging adults of mixed HIV serostatus and examining associations longitudinally. Moreover, future researchers should conduct multi-method studies, incorporating objective measures of stress and HRQoL to bolster these findings.

This study’s findings of moderation in the relationship between stress and HRQoL by personal mastery, emotional social support, and resilience among aging adults of mixed HIV serostatus, provide support to the contention that increased levels of psychosocial resources may improve physical HRQoL among PwH. Thus, interventions that focus on bolstering global perceptions of personal mastery may equip individuals with cognitive tools to use in the face of the myriad of physical challenges presented by living with HIV and aging. Furthermore, facilitating and encouraging the use of emotional social support and fostering high-quality social interactions may be protective of HRQoL. Taken together, these and other psychosocial resources may be particularly helpful in attenuating the negative effect of perceived stress subsumed by an HIV diagnosis, which might otherwise affect HRQoL.

## Data Availability

Available upon request.

## Code Availability

Available upon request.
